# PCSK9 Inhibitors Have Apolipoprotein C-III-Related Anti-Inflammatory Activity, Assessed by 1H-NMR Glycoprotein Profile in Subjects at High or very High Cardiovascular Risk

**DOI:** 10.3390/ijms24032319

**Published:** 2023-01-24

**Authors:** Pere Rehues, Josefa Girona, Montse Guardiola, Núria Plana, Roberto Scicali, Salvatore Piro, Ovidio Muñiz-Grijalvo, José Luis Díaz-Díaz, Lluís Recasens, Marta Pinyol, Roser Rosales, Yaiza Esteban, Núria Amigó, Lluís Masana, Daiana Ibarretxe, Josep Ribalta

**Affiliations:** 1 Universitat Rovira i Virgili, Departament de Medicina i Cirurgia, Unitat de Recerca en Lípids i Arteriosclerosi, 43201 Reus, Spain; 2 Institut d’Investigació Sanitària Pere Virgili, 43204 Reus, Spain; 3 Centro de Investigación Biomédica en Red de Diabetes y Enfermedades Metabólicas Asociadas, 28029 Madrid, Spain; 4 Unitat de Medicina Vascular i Metabolisme, Servei de Medicina Interna, Hospital Universitari Sant Joan de Reus, 43204 Reus, Spain; 5 Department of Clinical and Experimental Medicine, University of Catania, 95131 Catania, Italy; 6 Unidad Clinico-Experimental de Riesgo Vascular, Hospital Virgen del Rocío, 41013 Sevilla, Spain; 7 Department of Internal Medicine, Complejo Hospitalario Universitario A Coruña, 15006 A Coruña, Spain; 8 Heart Diseases Biomedical Research Group, IMIM (Hospital del Mar Medical Research Institute), 08003 Barcelona, Spain; 9 Cardiac Rehabilitation Unit, Department of Cardiology, Hospital del Mar, 08003 Barcelona, Spain; 10 Biosfer Teslab, 43201 Reus, Spain

**Keywords:** PCSK9, alirocumab, glycoproteins, apolipoprotein C-III, inflammation, LDL

## Abstract

**Highlights:**

What are the main findings?

- PCSK9 inhibition significantly reduces 1H-NMR glycoprotein signals and does not affect hsCRP levels.
- Apolipoprotein C-III and triglycerides are also decreased by iPCSK9.
- The decrease in glycoproteins correlates with the decrease in apoC-III and TG.

What is the implication of the main finding?

- PCSK9 inhibition significantly reduces inflammation

**Abstract:**

Atherosclerosis is a chronic inflammatory disease caused by the accumulation of cholesterol in the intima. Proprotein convertase subtilisin/kexin type 9 inhibitors (iPCSK9) can reduce low-density lipoprotein (LDL) cholesterol levels by 60%, but there is still no evidence that they can lower markers of systemic inflammation such as high-sensitivity C-reactive protein (hsCRP). Acute-phase serum glycoproteins are upregulated in the liver during systemic inflammation, and their role as inflammatory biomarkers is under clinical evaluation. In this observational study, we evaluate the effects of iPCSK9 on glycoproteins (Glyc) A, B and F. Thirty-nine patients eligible for iPCSK9 therapy were enrolled. One sample before and after one to six months of iPCSK9 therapy with alirocumab was obtained from each patient. Lipids, apolipoproteins, hsCRP and PCSK9 levels were measured by biochemical analyses, and the lipoprotein and glycoprotein profiles were measured by 1H nuclear magnetic resonance (1H-NMR). The PCSK9 inhibitor reduced total (36.27%, *p* < 0.001), LDL (55.05%, *p* < 0.001) and non-high-density lipoprotein (HDL) (45.11%, *p* < 0.001) cholesterol, apolipoprotein (apo) C-III (10%, *p* < 0.001), triglycerides (9.92%, *p* < 0.001) and glycoprotein signals GlycA (11.97%, *p* < 0.001), GlycB (3.83%, *p* = 0.017) and GlycF (7.26%, *p* < 0.001). It also increased apoA-I (2.05%, *p* = 0.043) and HDL cholesterol levels (11.58%, *p* < 0.001). Circulating PCSK9 levels increased six-fold (626.28%, *p* < 0.001). The decrease in Glyc signals positively correlated with the decrease in triglycerides and apoC-III. In conclusion, in addition to LDL cholesterol, iPCSK9 therapy also induces a reduction in systemic inflammation measured by 1H-NMR glycoprotein signals, which correlates with a decrease in triglycerides and apoC-III.

## 1. Introduction

Atherosclerosis, characterized as a chronic inflammatory disease of the large arteries [1,2], is the main underlying cause of cardiovascular diseases. Atherosclerotic cardiovascular disease (ASCVD) is considered the main cause of death worldwide and it has been clearly shown that LDL cholesterol lowering and anti-inflammatory therapies are effective in reducing ASCVD incidence [3,4,5,6,7]. Although statins exhibit important lipid-lowering and anti-inflammatory actions [8,9], one of the most intensive low density lipoprotein (LDL)-lowering agents are proprotein convertase subtilisin/kexin type 9 inhibitors (iPCSK9), which can reduce LDL cholesterol by 60% [10,11]. Despite the net clinical benefit of iPCSK9 on ASCVD risk reduction, in randomized clinical trials iPCSK9 has not demonstrated anti-inflammatory activity and failed to reduce high-sensitivity C-reactive protein (hsCRP) [12,13], a circulating inflammatory marker highly correlated with ASCVD and largely utilized in clinical practice [5,14,15].

However, recent in vivo and in vitro studies have found an association between PCSK9 and inflammation, independent of cholesterol levels. More specifically, in THP1 macrophages it has been shown that overexpression of PCSK9 upregulates the Toll-like receptor 4 (TLR4)/nuclear factor kappa B (NF-кB) proinflammatory pathway, a known contributor of atherosclerotic inflammation [16], and stimulation with PCSK9 enhances the expression of a set of proinflammatory chemokines and cytokines [17]. Moreover, in mice models, PCSK9 inhibition improved vascular inflammation and reduced the infiltration of immune cells in the plaque [18]; immunization against PCSK9 increased Th2 lymphocytes and interleukin (IL)—4 levels [19], and it has been suggested that iPCSK9 could suppress the chronic inflammatory state in atherosclerosis [20]. Overall, most in vitro and in vivo studies suggest that iPCSK9 may have anti-inflammatory effects, but other findings report an increased LPS uptake by the LDL receptor in endothelial cells after iPCSK9, which triggers the secretion of proinflammatory cytokines [21].

Several circulating inflammatory biomarkers, such as IL-6, IL-1β and tumor necrosis factor-α, have been associated with ASCVD risk [6,22,23], and some inflammation mediators are closely associated with lipoprotein metabolism such as the anti-inflammatory high-density lipoprotein (HDL) or the pro-inflammatory apolipoprotein (apo) C-III, the latter directly involved in monocyte adhesion and activation [24].

New biomarkers, such as plasma acute-phase glycoproteins, which can be detected by Nuclear Magnetic Resonance spectroscopy (1H-NMR) as glycoproteins A, B and F, have emerged as promising tools to detect inflammatory patterns in the onset and progression of ASCVD [25]. The 1H-NMR glycoproteins signals are a composite 1H-NMR response originated by the N-acetyl methyl structures on glycoproteins, mainly acute-phase plasma glycoproteins with antennary N-glycans [26] which are proportional to the plasmatic glycoprotein bonds. As assessed by 1H-NMR, three glycoprotein (Glyc) peaks can be detected (A, B and F) which are different signals of the same process. Unlike common biomarkers of inflammation, such as hsCRP or inflammatory cytokines, GlycA and GlycB are biomarkers that integrate the protein levels and the bond aggregation state of several of the most abundant acute-phase proteins in serum (α1-acid glycoprotein, haptoglobin, α1-antitrypsin, α1-antichymotrypsin, transferrin) and play a key role in inflammatory processes [27,28,29]. Some studies demonstrate that glycoproteins are very sensitive and robust biomarkers of low-grade inflammation in metabolic disorders and cardiovascular risk, as reported by analysis of large cohort studies, such as the JUPITER trial and the Women’s Health Study, where GlycA was associated with incident cardiovascular events [30,31].

In this observational study, we aimed to evaluate the anti-inflammatory capacity of iPCSK9 on a glycoprotein profile evaluated by 1H-NMR in a cohort of subjects at high or very high cardiovascular risk.

## 2. Results

The study population consisted of 39 patients with dyslipidemia who were eligible for PCSK9 inhibition therapy. The mean age of the participants was 56.15 ± 10.5 years old. The study cohort included 12 patients with diabetes (30.8%) and 25 with arterial hypertension (64.1%). Thirty-four patients were receiving statin therapy (87.2%), and 26 were on ezetimibe therapy (66.7%) (Table 1).

### 2.1. Changes in Inflammation: Glycoproteins

**Figure 1:**
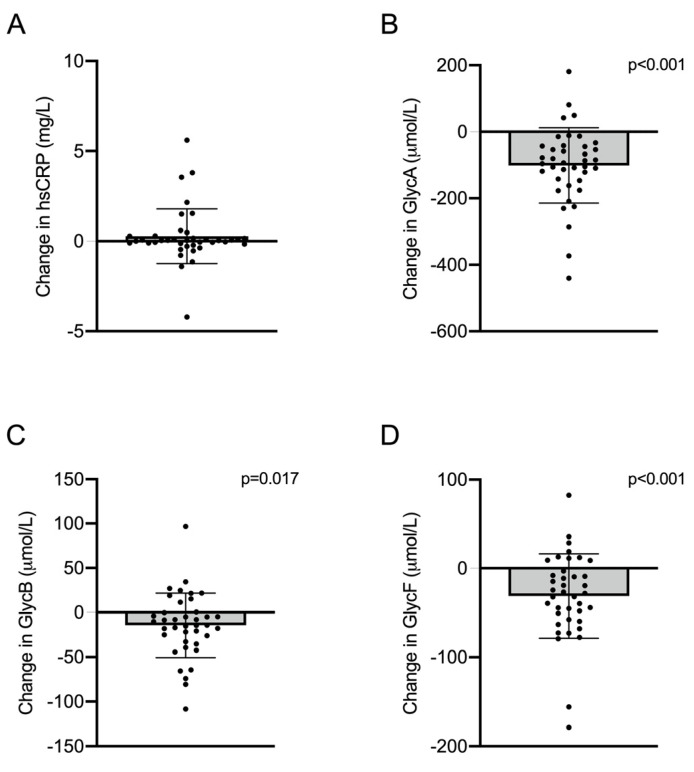
Mean changes in (**A**) high-sensitivity C-reactive protein (hsCRP) and 1H nuclear magnetic resonance (1H-NMR)—assessed glycoproteins (**B**) A, (**C**) B and (**D**) F concentrations after iPCSK9 therapy. Error bars indicate SD.

Indicators of systemic inflammation were measured before and after PCSK9 inhibition therapy. No significant changes in hsCRP levels were observed; however, all Glyc signals (GlycA, GlycB, and GlycF) were decreased after the treatment. Specifically, reductions of 11.97% (*p* < 0.001), 3.83% (*p* = 0.017) and 7.26% (*p* < 0.001) were observed for GlycA, GlycB and GlycF, respectively (Figure 1 and Supplemental Figure S1). Changes in GlycA and GlycF, but not GlycB, were negatively correlated with basal levels of GlycA and GlycF, respectively, and after adjusting for confounding variables (Supplemental Figure S2C–E).

**Figure 2:**
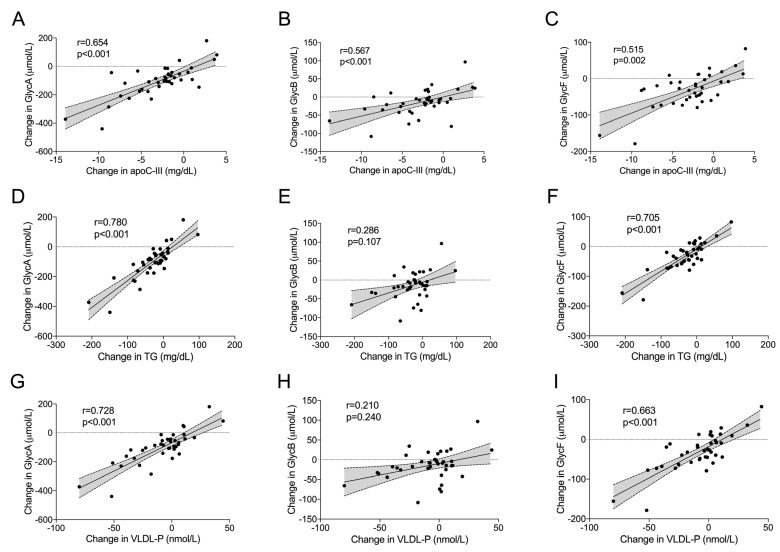
Correlation between change in 1H nuclear magnetic resonance (1H-NMR)—assessed glycoproteins (Glyc) and change in plasma concentrations of lipids, apolipoproteins (apo) and 1H-NMR—assessed lipoproteins. (**A**–**C**), Correlations between changes in Glyc concentrations and changes in apoC-III plasma concentrations. (**D**–**F**), Correlations between changes in Glyc concentrations and changes in plasma triglycerides (TG). (**G**–**I**), Correlations between changes in Glyc concentrations and changes in VLDL particle concentrations (VLDL-P). Lines represent linear regression with 95% CI.

To understand such a decrease in inflammatory markers, the association between changes in glycoprotein signals (GlycA, GlycB and GlycF) and changes in other biochemical measures was evaluated. Changes in all Glyc signals were strongly and directly correlated with changes in triglycerides and apoC-III; GlycA and GlycF also correlated with changes in total VLDL particle concentrations (GlycB showed a nonsignificant trend). After controlling for confounding variables (sex, age, body mass index, arterial hypertension, statin dose and PCSK9 inhibitor dose), all correlations were still significant except the association between change in GlycB and change in triglycerides (Figure 2). Among these, change in apoC-III was the variable with the strongest correlation, with changes in GlycB, while changes in triglycerides was the most strongly correlated variable with change in GlycF and GlycA. Specifically, all individuals who had a decrease in triglyceride concentrations after the treatment also had a decrease in GlycA levels.

### 2.2. Changes in Lipids, Lipoproteins and Apolipoproteins

Table 2 shows lipid, apolipoprotein and other biochemical changes after PCSK9 inhibition therapy. In all patients, iPCSK9 treatment significantly reduced the plasma concentrations of total and LDL cholesterol by 36.27% (*p* < 0.001) and 55.05% (*p* < 0.001), respectively. ApoB (43.27%, *p* < 0.001) and Lp(a) (21.98%, *p* < 0.001) levels were also reduced. HDL cholesterol and apoA-I levels significantly increased by 11.58% (*p* < 0.001) and 2.05% (*p* = 0.043), respectively.

Plasma triglyceride levels and apoC-III were also significantly decreased after iPCSK9 therapy, with mean reductions of 9.92% (*p* < 0.001) and 10% (*p* < 0.001), respectively (Table 2). Changes in triglyceride concentrations showed a strong negative correlation with basal triglycerides (Supplemental Figure S2A).

### 2.3. Changes in Lipoprotein Size and Number

As expected, a marked decrease in particle number was observed in all LDL subclasses (Table 3). Medium LDL particles showed larger differences after PCSK9 inhibition (58.97% reduction, *p* < 0.001), followed by large and small LDL particles (34.06%, *p* < 0.001; and 26.4%, *p* < 0.001 reduction, respectively). A significant increase in total HDL particles was also observed (6.79%, *p* = 0.006), which was due to an increase in the small HDL subtype (15.29%, *p* < 0.001), as large and medium HDL particle concentrations were actually decreased (9.24%, *p* < 0.001, and 5.71%, *p* < 0.001 lower than before therapy, respectively). No significant changes in VLDL particle concentrations were observed.

The mean size of LDL and HDL particles was only slightly decreased after treatment by 1.1% (*p* < 0.001) and 1.2% (*p* < 0.001), respectively, whereas VLDL diameter was unchanged (Table 3).

### 2.4. Changes in Circulating PCSK9

**Figure 3:**
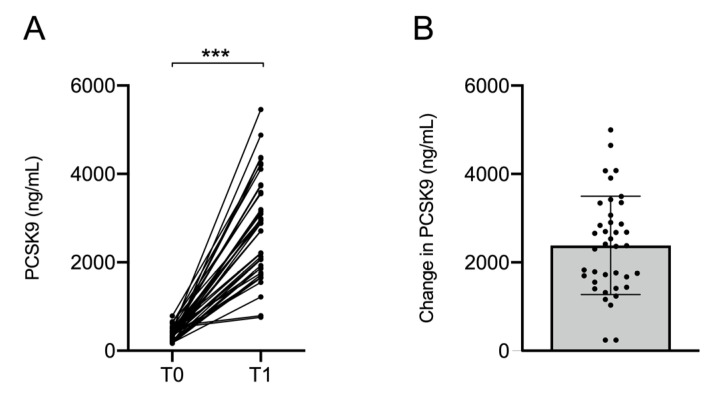
Circulating PCSK9 concentrations before and after PCSK9 inhibition therapy. (**A**) Individual patient data and (**B**) mean change in PCSK9 concentration. Error bars indicate SD. *** *p* < 0.001.

After treatment with iPCSK9, an increase in plasma total PCSK9 levels was noted as levels were approximately six-fold higher in the posttreatment measure (626.28% increase, Figure 3). This increase was higher in patients receiving 150 mg of alirocumab (844.32%) compared with those receiving 75 mg of alirocumab (519.81%) (*p* = 0.026). Patients who had received six months of treatment also had a higher increase in PCSK9 levels (930.54%) compared with those who had only one month of treatment at the time of the study (545.76%) (*p* = 0.009).

The increase in circulating PCSK9 levels showed a strong and inverse correlation with differences in total cholesterol (r = −0.633, *p* < 0.001), LDL cholesterol (r = −0.732, *p* < 0.001), apoB (r = −0.654, *p* < 0.001), total LDL particles (r = −0.531, *p* = 0.001) and all subtypes of LDL particles (large: r = −0.513, *p* = 0.002; medium: r = −0.540, *p* = 0.001; and small: r = −0.439, *p* = 0.011) and an inverse association with changes in apoA-I levels (r = 0.374, *p* = 0.032). No significant correlations were found between the increase in PCSK9 concentrations and changes in GlycA (r = −0.193, *p* = 0.282), GlycB (r = 0.184, *p* = 0.305) or GlycF (r = −0.087, *p* = 0.632).

### 2.5. ApoC-III Concentrations in Lipoprotein Fractions

Given that we observed a significant reduction in plasma total apoC-III, as well as a significant correlation with changes in glycoproteins, we measured apoC-III concentrations in each lipoprotein fraction before and after the treatment in a subgroup of 16 patients. Multiplex analysis of the apoC-III concentrations revealed a significant 46% reduction in apoC-III in the LDL fraction. The 9% reduction in apoC-III in the VLDL fraction was not statistically significant.

## 3. Discussion

In this prospective observational study, we explored changes in inflammatory biomarkers after iPCSK9 therapy and their relation with changes of lipoproteins and apolipoproteins. We showed that glycoproteins A, B and F were significantly decreased after PCSK9 inhibition therapy, and these effects were associated with the changes in total triglycerides and apoC-III, thus suggesting that the anti-inflammatory activity of iPCSK9 is modulated by triglyceride metabolism. 

### 3.1. Changes in Inflammatory Markers

The CANTOS study clearly identified inflammation as one of the key biological processes to prevent atherosclerosis in addition to lowering LDL [5]. Therefore, a topic of clear clinical interest was to understand whether iPCSK9 therapy could exhibit an anti-inflammatory benefit in addition to its lipid-lowering action. In this context, we tested a set of biomarkers including hsCRP, GlycA, GlycB and GlycF and we observed a reduction of all glycoproteins; in particular, GlycA and GlycF showed a highly significant reduction of approximately 12% and 7%, respectively. Our results confirmed the extensive previous data [32] indicating that iPCSK9 reduction of LDL cholesterol was not accompanied by a decrease of hsCRP.

Glycosylation plays a key role in inflammation, and reduced levels of these parameters indicate a lower systemic inflammatory state in the treatment group. GlycA and GlycF are highly covariant and are associated with the presence of plasmatic N-acetylgalactosamine, and N-acetylglucosamine bound (GlycA) and unbound (GlycF) to proteins. Most of the methodological approaches do not distinguish between these two 1H-NMR signals, as they appear in the same spectral region and are simultaneously determined contributing into the total GlycA area [33]. Previous studies reported an association between GlycA and a higher risk of CVD development independently associated with lipid levels [34,35], implying a higher number of N-acetylglucosamine residues present in plasmatic proteins in several inflammatory based conditions [27,36,37]. On the other hand, the study group showed a significant but modest reduction in sialylation, up to a 3.83 % reduction in GlycB levels after treatment. The different magnitude of the effect on the GlycA/GlycF compared to the GlycB levels may reflect their differential glycosylation sensitivity associated with the treatment. This response difference is consistent with the specific variable variances, as the GlycA variance doubles the GlycB variance when these two distributions are compared [35,38]. The 1H-NMR is not sensitive enough to distinguish between different antennary structures, but it could be possible that the structurally different glycans containing neuraminic acid are differently modified after treatment. Other studies have evaluated the effect of different drugs associated with 1H-NMR visible glycoprotein markers. Except for statins treatment [39], the effects of different drugs monitoring inflammatory status generally showed a reduction of glycosylated parameters: when using Anti-TNF and monoclonal antibodies for inflammatory based conditions [40], using antiretroviral treatments [41], metformin [42] or probiotics [43].

To the best of our knowledge, this is the first study reporting significant reductions in glycoproteins after iPCSK9 therapy. In light of the study by Sliz et al. reporting a nonsignificant and very modest GlycA reduction in carriers of PCSK9 rs11591147 to mimic PCSK9 therapeutic inhibition [44], our results show that PCSK9 inhibitors display a more intense anti-inflammatory capacity. Although glycoproteins reflect iPCSK9 anti-inflammatory activity, hsCRP does not. This finding likely relates to their characteristics as biomarkers; while CRP is a single protein with high intraindividual variability, GlycA is a composite biomarker that integrates the protein levels and glycosylation states of several of the most abundant acute phase proteins in circulation, and shows lower intraindividual variability. Numerous studies have demonstrated the utility of GlycA as a biomarker for cardiometabolic risk. Interestingly, in most cases, the associations are not related to CRP, suggesting that CRP and GlycA reflect different aspects of the inflammatory status (reviewed in [45]) and are associated with different cardiovascular outcomes [46].

As we observed reductions in several lipids and apolipoproteins other than LDL cholesterol following iPCSK9 treatment, we explored whether changes in glycated proteins could be related to changes in other lipoproteins or apolipoproteins. We found a highly significant correlation between decreases in all three glycoproteins, and decreases in triglyceride rich lipoproteins, remnant cholesterol and apoC-III. Triglyceride-rich lipoproteins and remnant cholesterol are key drivers of cholesterol accumulation in myocardial infarction and incident cardiovascular disease [47,48], and genetic and therapeutic reduction of apoC-III significantly reduces the incidence of cardiovascular disease [49,50]. In particular, a significant reduction of apoC-III bound to LDL after treatment has been observed, as apoC-III–enriched LDL displays an increased capacity to bind to the extracellular matrix [51]. Altogether, our results show a global antiatherogenic effect of PCSK9 inhibition beyond that of LDL reduction.

### 3.2. Changes in Lipids, Lipoproteins and Apolipoproteins

Alirocumab treatment resulted in a global improvement of the lipid profile, defined by a 55% reduction in LDL cholesterol, a 10% reduction in plasma triglycerides and a 12% increase in HDL cholesterol. Additionally, apoB, apoC-III and Lp(a) were reduced by 43%, 10% and 22%, respectively. These changes are consistent with those reported in a pooled analysis of 10 phase III alirocumab trials with 4983 participants [52]. Beyond the reductions in LDL cholesterol, decreases in remnant cholesterol and apoC-III, which likely occur as a consequence of a reduction in plasma triglycerides, are important from an anti-atherogenic point of view. While remnant cholesterol has been identified as a more determinant factor than LDL cholesterol in causing incident cardiovascular disease [47] and myocardial infarction [48], apoC-III is a functionally complex protein involved in several atherogenic processes, such as delayed lipoprotein removal, impaired triglyceride hydrolysis and increased adherence to the extracellular matrix. In this context, the abovementioned 46% reduction in apoC-III bound to LDL detected after treatment is noteworthy. Moreover, apoC-III is also involved in pancreatic dysfunctions leading to diabetes [53]. The mechanisms by which triglycerides and remnant cholesterol are associated with cardiovascular disease may include the production of proinflammatory cytokines, excessive release of free fatty acids, impairment of coagulation factors, and fibrinolysis [54]. Additionally, excess free fatty acids result in increased triglycerides, both having an inflammatory effect [55].

NMR lipoprotein analysis allowed us to assess how changes in lipids and the lipid content of lipoproteins actually translated into changes in the concentration of each lipoprotein subclass. The 10% reduction in plasma triglycerides did not result in significant changes in the concentration of VLDL particles of either size, suggesting increased triglyceride hydrolysis rather than decreased secretion or increased clearance of VLDL particles. Although previous studies reported reductions in VLDL particle concentration after iPCSK9 therapy [56,57], a closer look at our population reveals high interindividual variability, which probably explains the absence of an average change. Conversely, the 55% reduction in LDL cholesterol resulted in a significant reduction of all LDL particles, including large (−34%), medium (−59%) and small (−26%), in accordance with previous studies [56,57]. In the study by Koren et al. [57], alirocumab treatment shifted the HDL-P profile from small to large. However, our results indicate the opposite trend, with a 7% elevation of total HDL particles, mostly due to the small fraction. Although some studies report that small high-density lipoprotein (HDL) particles are effective vehicles for reverse cholesterol transport [58], this is a controversial issue, and more research needs to be conducted to better characterize HDL subspecies, both relative to size and protein content, in relation to their functionality [44].

### 3.3. PCSK9 Plasma Concentration

The concentration of total PCSK9 after treatment increased an average of six-fold, and this increase was strongly inversely correlated with LDL cholesterol and LDL particle concentration. These observed effects of alirocumab on lipoprotein particles compare with previous NMR analyses, showing that circulating levels of free PCSK9 correlate with LDL particle concentration [59] and confirming that these increases in PCSK9 concentration are compatible with a global improvement in atherogenic biomarkers. In addition, other studies proposed that the change in PCSK9 concentration could be used to confirm adherence to inhibitor therapy [60]. Increases in PCSK9 concentration did not correlate with changes in glycoproteins, suggesting that the changes were not associated.

One limitation of this study may be the differences in iPCSK9 and statin doses and follow-up time. PCSK9i dose and statin dose has been considered a confounder variable, and no significant differences in the variables of interest were observed between groups of patients at different follow-up times; however, further studies with a more homogenous group of patients and intervention would be of interest to confirm these findings. Since we report beneficial effects of iPCSK9 on systemic inflammation, different to those associated with hsCRP, further mechanistic studies will be necessary to understand the benefits of this anti-inflammatory action on atherosclerosis progression and ASCVD, and on other inflammatory conditions such as diabetes, systemic lupus erythematosus or rheumatoid arthritis.

To summarize, in the present study we show that, in addition to LDL cholesterol, ApoB and Lp(a), iPCSK9 also decreases plasma apoC-III, triglycerides and circulating biomarkers of inflammation: GlycA, B and F. Conversely, no reductions in hsCRP are observed. HDL cholesterol and ApoA-I are slightly increased after treatment and a six-fold increase in circulating PCSK9 levels is also observed. In conclusion, we show how glycoproteins reveal significant anti-inflammatory activity of iPCSK9, which seems to be strongly related to triglycerides and apoC-III.

## 4. Material and Methods

### 4.1. Design and Study Subjects

Patients who underwent PCSK9 inhibition were enrolled from 5 centers: Hospital Universitari Sant Joan in Reus, Hospital Abente y Lago in A Coruña, Hospital Virgen del Rocío in Sevilla, Hospital del Mar in Barcelona and the Lipid Centre of the Garibaldi Hospital/University of Catania, Sicily, Italy. All subjects started PCSK9 inhibitor treatment according to the current guidelines for the treatment of dyslipidemia of the European Atherosclerosis Society and the European Society of Cardiology [61].

Anamnesis of the patients, including medical history and treatment, anthropometric data and blood pressure, were collected. A blood sample was obtained from each patient after overnight fasting, before initiation of iPCSK9 therapy with alirocumab and after 1 to 6 months of treatment. Plasma aliquots were prepared for immediate storage at −80 °C in the Biobank at each center prior to use. Standard biochemical analyses, PCSK9 plasma concentrations and full lipoprotein and glycoprotein profiles assessed by 1H-NMR were performed pre- and post-PCSK9 inhibition therapy. Lipoprotein isolation by ultracentrifugation was also performed in a subgroup of patients. Patients received either 75 mg or 150 mg alirocumab subcutaneously every 2 weeks.

This study was approved by the Ethical and Clinical Investigation Committee of the Institut d’Investigació Sanitària Pere Virgili (IISPV) and fulfilled the principles of the Helsinki Declaration. A written consent form was signed by all participants.

### 4.2. Clinical and Standard Biochemical Analysis

Anamnesis and anthropometric data, including sex, age, clinical history and medication, were recorded and included in the research institute database. BMI was calculated from weight and height measurements (kg/m^2^). Standard biochemical parameters, including lipids, apolipoproteins, Lp(a), blood glucose and hsCRP, were measured using colorimetric, enzymatic and immunoturbidimetric assays (Spinreact, Girona, Spain; Horiba, Kioto, Japan), which were adapted for automation by clinical chemistry Cobas Mira Plus Autoanalyzer (Roche Diagnostics, Basilea, France).

The standard LDL cholesterol concentration was calculated using the Friedewald formula [62], and the remnant cholesterol concentration was calculated by subtracting LDL and HDL cholesterol from total cholesterol.

Circulating PCSK9 was measured by enzyme-linked immunosorbent assay (ELISA) kits (R&D Systems, Minneapolis, MN, USA) following the reagent manufacturer’s instructions.

### 4.3. Lipoprotein Particle Analysis by 1H-NMR

The lipoprotein particle number and size were assessed using the Liposcale test^®^ by Biosfer Teslab SL, which is a new generation 2D-1H-NMR test developed in collaboration with our group [63]. In brief, 200 µL of serum was diluted with 50 µL of deuterated water and 300 µL of 50 mM phosphate-buffered solution (PBS) at pH 7.4. The 1H-NMR spectra were recorded at 310 K on a Bruker Avance III 600 spectrometer operating at a proton frequency of 600.20 MHz (14.1 T). The particle size and particle number concentration of VLDL, LDL and HDL (including total and large, medium and small subtypes of each lipoprotein type) were analyzed. Particle concentrations and diffusion coefficients were obtained from the measured distinct methyl groups of the 2D 1H-NMR spectra after the deconvolution analysis of the signals of the 1H-NMR pulse. The variation coefficients for particle number were between 2% and 4%. The variation coefficients for particle size were less than 0.3%.

### 4.4. Glycoprotein Analysis by 1H-NMR

Glycoprotein profiling in plasma samples was analyzed by 1H-NMR as previously described [29]. The region of the 1H-NMR spectrum where the glycoproteins resonate (2.15–1.90 ppm) was analyzed using several functions according to the chemical shift: GlycA, GlycB and GlycF. For each function, we determined the total area and transformed the total area to concentration according to the number of sugar–protein bonds. The area, height, position, and bandwidth were also calculated. The area of GlycA provides the concentrations of acetyl groups from protein-bound N-acetylglucosamine and N-acetylgalactosamine, the area of GlycB provides concentrations of acetyl groups from N-acetylneuraminic acid in plasma proteins, and the area of GlycF reflects the concentration of free acetyl groups N-acetylglucosamine, N-acetylgalactosamine, and N-acetylneuraminic acid in the sample, as reported in [35].

### 4.5. Lipoprotein Isolation by Ultracentrifugation and Apolipoprotein Analysis

To determine apoC-III cargo, lipoproteins (VLDL, IDL, LDL and HDL) were isolated using sequential preparative ultracentrifugation according to previously described techniques [64] in a subgroup of patients (*n* = 16). Ultracentrifugation was performed in a Beckmann Type 50.4 Ti Fixed-Angle Rotor in an Optima XPN100 ultracentrifuge (Beckman Coulter, Indinapolis, IN, USA) at 37,000 rpm for 20 h at 4 °C. The apoC-III content of each lipoprotein subfraction was determined using an APOMAG-62K kit (Millipore, Burlington, MA, USA).

### 4.6. Statistical Analysis

The sample size was calculated based on the expected difference in inflammatory markers. The available data show that there is no effect of iPCSK9 on CRP levels, so we would not expect large effects on GlycA levels. Therefore, to detect a 10% difference in GlycA levels after iPCSK9 treatment, a minimum of 25 participants was required.

Statistical analyses were performed using IBM SPSS Statistics^®^ for Windows, version 28 (IBM Corp., Armonk, NY, USA). Normality of variables was assessed using the Shapiro-Wilk test; comparisons between pre- and posttreatment data were performed using paired t-tests if both variables were normally distributed, and Wilcoxon signed-rank tests if otherwise. Correlation coefficients were calculated using Pearson (for normally distributed variables) or Spearman rank correlation (for nonnormally distributed variables) and adjusted for sex, age, body mass index, statin dose, hypertension and PCSK9 inhibitor dose.

## Figures and Tables

**Table 1:** Characteristics of the study population.

Patients	39
Women	14 (35.9)
Smoker	
No	16 (41.0)
Yes	10 (25.6)
Ex-smoker	13 (33.3)
Arterial hypertension	25 (64.1)
Type 2 diabetes	12 (30.8)
Dyslipidemia	38 (97.4)
Type of dyslipidemia	
HFH	19 (48.7)
Polygenic FH	3 (7.7)
FCH	2 (5.13)
Other	14 (35.9)
CAD	21 (53.8)
Stroke	4 (10.3)
PAD	2 (5.1)
Statins	34 (87.2)
Statin type and dose (in mg)	
Pr40, F80, S20, A10, P1	3 (7.7)
S40, A20, R5, P2	1 (2.6)
A40, R10, P4	11 (28.2)
A80, R20	18 (46.2)
R40	1 (2.6)
Ezetimibe	26 (66.7)
Fibrates	2 (5.1)
Antiaggregants	24 (61.5)
Anticoagulants	1 (2.6)
Age, years	56.15 ± 10.50
Weight, kg	78.32 ± 15.41
Height, cm	166.62 ± 9.43
BMI, kg/m^2^	28.08 ± 4.26
Systolic BP, mm Hg	130.00 (120.00–143.75)
Diastolic BP, mm Hg	78.18 ± 9.54
hsCRP, mg/L	0.54 (0.18–1.20)

Data are presented as mean ± SD, median (interquartile range) or number of cases (percentage). Abbreviations: HFH, heterozygous familial hypercholesterolemia; FH, familial hypercholesterolemia; FCH, familial combined hyperlipidemia; CAD, coronary artery disease; PAD, peripheral artery disease; Pr, pravastatin; F, Fluvastatin; S, simvastatin; A, atorvastatin; P, pitavastatin; R, rosuvastatin; BMI, body mass index; BP, blood pressure; hsCRP, high-sensitivity C-reactive protein.

**Table 2:** Biochemical profile.

	Pre-Treatment	Post-Treatment	Percent Change	*p*-Value
Lipids and apolipoproteins				
Total cholesterol, mg/dL	188.42 (171.52–248.65)	120.08 (106.85–153.76)	−36.27	<0.001
Triglycerides, mg/dL	115.94 (97.13–171.91)	104.43 (90.49–134.96)	−9.92	<0.001
LDL cholesterol, mg/dL	118.23 (104.89–174.87)	53.14 (35.93–84.72)	−55.05	<0.001
HDL cholesterol, mg/dL	40.35 (33.23–48.31)	45.02 (38.65–51.81)	11.58	<0.001
Remnant cholesterol, mg/dL	23.19 (19.43–34.38)	20.89 (18.10–26.99)	−9.92	<0.001
non-HDL cholesterol, mg/dL	144.21 (128.66–204.51)	79.15 (64.29–109.56)	−45.11	<0.001
Lp(a), mg/dL	27.30 (9.95–78.53)	21.30 (7.52–62.75)	−21.98	<0.001
ApoB, mg/dL	104.00 (92.00–122.75)	59.00 (45.50–77.00)	−43.27	<0.001
ApoA-I, mg/dL	146.00 (124.50–174.75)	149.00 (131.50–189.75)	2.05	0.043
ApoC-III, mg/dL	9.00 (6.58–14.28)	8.10 (5.20–10.35)	−10	<0.001
HDL triglycerides, mg/dL	16.93 ± 5.46	16.09 ± 4.82	−4.99	0.254
HDL apoC-III, mg/dL	5.61 (3.91–10.26)	5.28 (2.50–8.25)	−5.88	0.087
Inflammatory markers				
hsCRP, mg/L	0.54 (0.18–1.20)	0.44 (0.23–1.54)	−18.52	0.492
GlycA, μmol/L	843.71 ± 154.98	742.68 ± 106.97	−11.97	<0.001
GlycB, μmol/L	376.82 ± 48.34	362.38 ± 48.12	−3.83	0.017
GlycF, μmol/L	229.95 (211.70–271.77)	213.26 (190.26–231.71)	−7.26	<0.001
PCSK9				
pcsk9, ng/mL	380.58 ± 148.70	2764.11 ± 1131.26	626.28	<0.001

Concentrations are presented as the mean ± SD or median (IQR). Percent changes are calculated from pretreatment and posttreatment mean or median values. Abbreviations: LDL, low-density lipoprotein; HDL: high-density lipoprotein; Lp(a), Lipoprotein (a); apo, apolipoprotein; hsCRP, high-sensitivity C-reactive protein; PCSK9, proprotein convertase subtilisin/kexin type 9.

**Table 3:** Lipoprotein particle number and size.

	Pre-Treatment	Post-Treatment	Percent Change	*p*-Value
VLDL-P, nmol/L	40.29 (34.70–67.25)	42.91 (37.84–57.99)	6.5	0.101
Large VLDL-P, nmol/L	1.22 (0.97–1.63)	1.44 (1.17–1.64)	18.03	0.179
Medium VLDL-P, nmol/L	4.61 (3.67–6.83)	4.62 (3.56–5.73)	0.22	0.138
Small VLDL-P, nmol/L	36.25 (28.83–58.34)	37.29 (32.12–50.01)	2.87	0.093
LDL-P, nmol/L	1510.75 (1318.89–1736.34)	936.07 (799.70–1108.24)	−38.04	<0.001
Large LDL-P, nmol/L	212.23 ± 41.99	139.94 ± 32.61	−34.06	<0.001
Medium LDL-P, nmol/L	443.57 (345.80–526.54)	182.00 (140.02–281.48)	−58.97	<0.001
Small LDL-P, nmol/L	855.14 (760.26–906.30)	629.38 (544.00–712.05)	−26.4	<0.001
HDL-P, μmol/L	25.34 ± 4.98	27.06 ± 6.32	6.79	0.006
Large HDL-P, μmol/L	0.30 ± 0.04	0.27 ± 0.03	−9.24	<0.001
Medium HDL-P μmol/L	9.28 (8.79–10.24)	8.75 (8.23–9.45)	−5.71	<0.001
Small HDL-P, μmol/L	15.45 ± 4.48	17.82 ± 5.91	15.29	<0.001
VLDL size, nm	42.19 ± 0.20	42.26 ± 0.17	0.16	0.065
LDL size, nm	21.11 ± 0.24	20.87 ± 0.28	−1.1	<0.001
HDL size, nm	8.34 (8.25–8.44)	8.24 (8.18–8.37)	−1.2	<0.001

Concentrations are presented as the mean ± SD or median (IQR). Percent changes are calculated from pretreatment and posttreatment mean or median values. Abbreviations: VLDL, very low-density lipoprotein; LDL, low-density lipoprotein; HDL, high-density lipoprotein; VLDL-P, VLDL particles; LDL-P, LDL particles; HDL-P, HDL particles.

## Data Availability

The data presented in this study are available on request from the corresponding author. Data will be publicly available once all the related ongoing analyses are completed.

## Supplementary Materials

The following supporting information can be downloaded at: https://www.mdpi.com/article/10.3390/ijms24032319/s1.

## Abbreviations

iPCSK9	proprotein convertase subtilisin/kexin type 9 inhibitors
VLDL	very low-density lipoprotein
LDL	low-density lipoprotein
HDL	high-density lipoprotein
Lp(a)	lipoprotein (a)
Apo	apolipoprotein
Glyc	glycoprotein
1H-NMR	1H nuclear magnetic resonance
hsCRP	high-sensitivity C-reactive protein
IL	interleukin
