# Supplementary material for: PCSK9 Inhibitors Have Apolipoprotein C-III-Related Anti-Inflammatory Activity, Assessed by 1H-NMR Glycoprotein Profile in Subjects at High or very High Cardiovascular Risk
Source: Int J Mol Sci. 2023 Jan 24;24(3):2319. doi: 10.3390/ijms24032319 (PMC9917120; doi:10.3390/ijms24032319)
Supplement: Supplementary file 1 [file ijms-24-02319-s001.zip › ijms-2126840-supplementary.pdf]

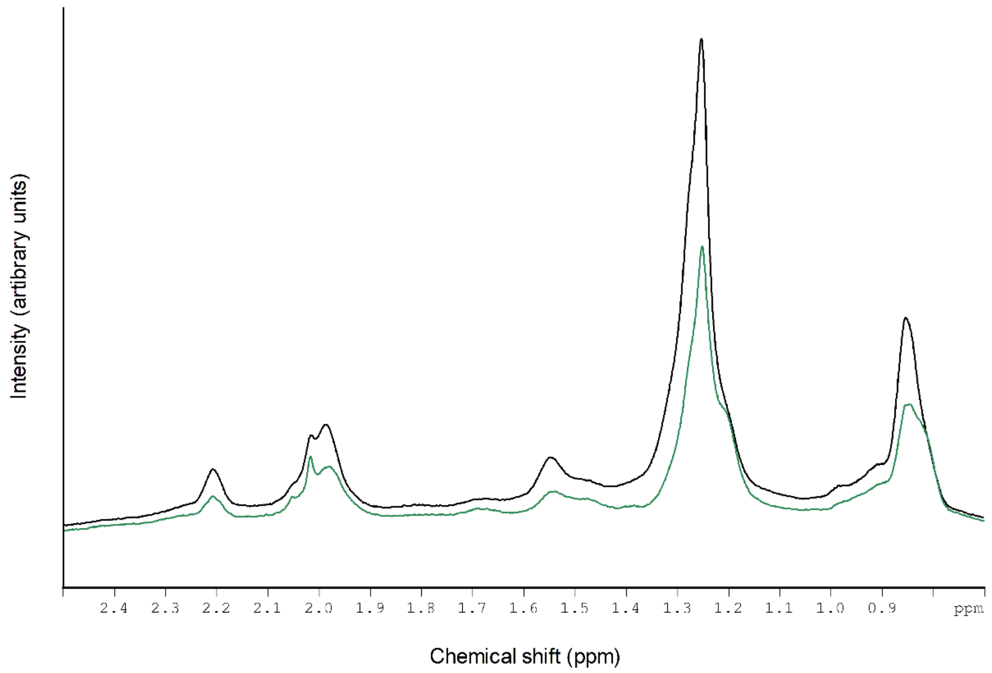

Figure S1. 1H-NMR spectra from one representative subject before (black) and after (green) iPCSK9 therapy. Glycoproteins resonate between 2.15 and 1.90 ppm.

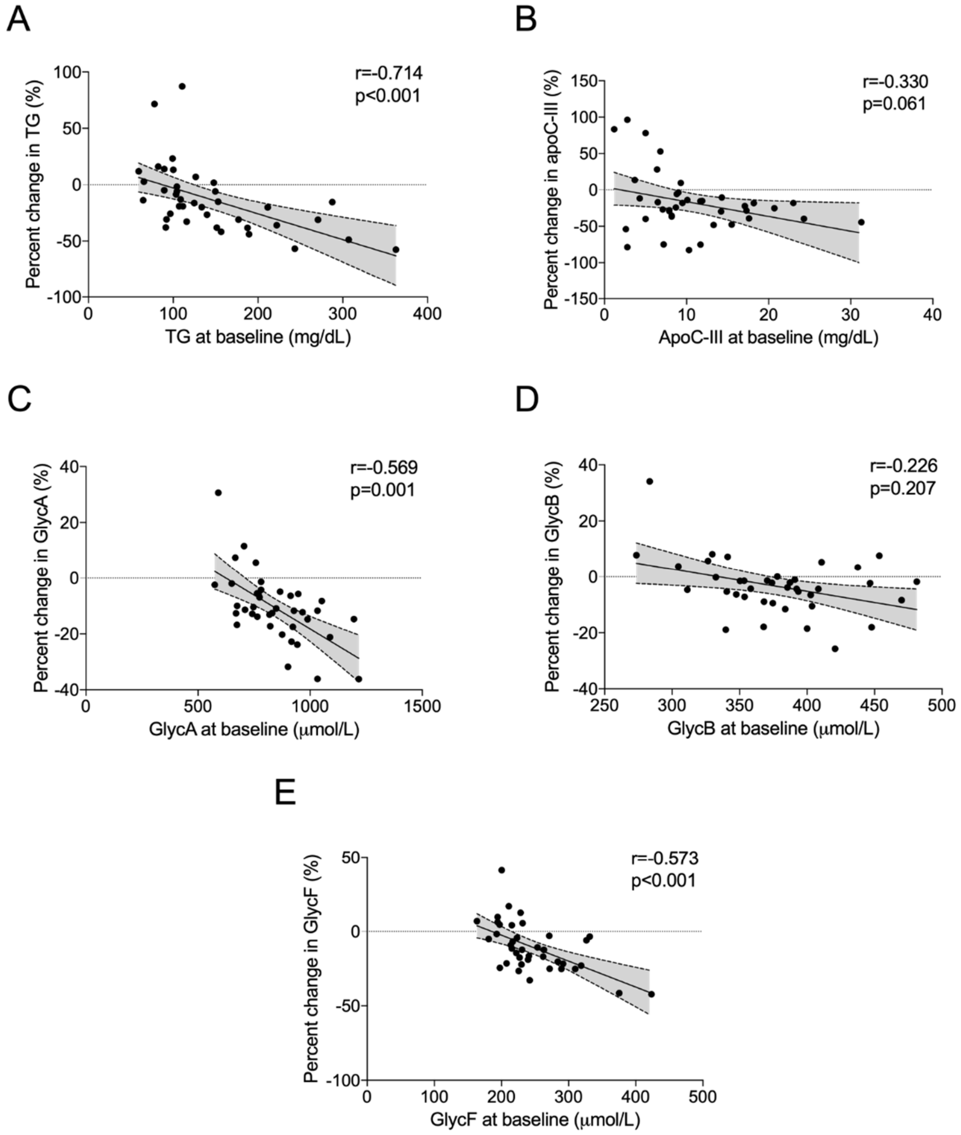

Figure S2. Correlation between percent change and basal concentration of (A) triglycerides (TG), (B) apoC-III and (C-E) Glyc signals. Lines represent linear regression with 95% CI.
